# The Interpersonal Mindfulness in Parenting Scale in Mothers of Children and Infants: Factor Structure and Associations With Child Internalizing Problems

**DOI:** 10.3389/fpsyg.2020.633709

**Published:** 2021-02-03

**Authors:** Virginia Burgdorf, Marianna Szabó

**Affiliations:** School of Psychology, The University of Sydney, Sydney, NSW, Australia

**Keywords:** IMP, mindful parenting, psychometric properties, experiential avoidance, parental beliefs, parental accommodation, child internalizing, children and infants

## Abstract

**Objectives:** Mindful parenting, measured by the Interpersonal Mindfulness in Parenting scale (IMP), is beneficial for parents and children. However, the IMP has not been validated in English-speaking parents. Further, little is known about whether mindful parenting is similar in parents of children vs. infants, or how it reduces child internalizing problems. We sought to validate the IMP in English-speaking mothers of children and infants, and to examine relationships between the facets of mindful parenting, child internalizing problems and parent variables related to internalizing.

**Methods:** Using confirmatory factor analyses, we examined the fit of various models of mindful parenting in English-speaking community-recruited mothers of children aged 3–18 years (*n* = 396) and infants aged 0–2 years (*n* = 320). We used regression analyses to investigate relationships between the facets of mindful parenting, child internalizing problems, and parent variables including parental experiential avoidance, unhelpful beliefs about child anxiety and accommodation of child anxiety.

**Results:** Mindful parenting can be measured in English-speaking mothers, using either a 5- or 6-factor, 29-item version of the IMP. These versions of the IMP operate similarly for mothers of children and infants. Child internalizing problems and related parent variables were best predicted by non-judgmental acceptance of parenting in mothers of children, and emotional self-awareness and non-reactivity in mothers of infants.

**Conclusions:** The IMP is a valid measure of mindful parenting in English-speaking mothers of children and infants. Mindful parenting predicts child internalizing problems and related parent variables, suggesting that mindful parenting programs could benefit families of children with internalizing problems, potentially by reducing parental experiential avoidance, unhelpful beliefs about or accommodation of child anxiety.

## Introduction

Mindful parenting has been defined as parenting with the aim of paying non-judgmental, non-reactive attention to each moment and interaction with the child (Kabat-Zinn and Kabat-Zinn, 1997). Mindful parents are thought to be able to regulate their parenting behaviors to better support their child's needs (Duncan et al., 2009). Indeed, a recent meta-analysis has shown that mindful parenting interventions are associated with reductions in parenting stress and children's externalizing and internalizing problems (Burgdorf et al., 2019). However, the mechanisms through which mindful parenting programs benefit parents and children are still largely unexplored, particularly in relation to child internalizing problems. To understand these mechanisms, a valid and reliable measurement of the dimensions of mindful parenting is necessary. The Interpersonal Mindfulness in Parenting scale (IMP; Duncan, 2007; Duncan et al., 2009) is the most widely used instrument for that purpose. However, the IMP was originally developed for parents of adolescents (Duncan, 2007) and it has been investigated primarily in relation to child externalizing behaviors (e.g., Haydicky et al., 2015). To date, very little is known about the psychometric properties of the IMP in mothers of infants, or its relationship with parenting behaviors related to child internalizing problems. This study aimed to contribute to a better understanding of these issues.

The first instrument developed to measure the construct of mindful parenting was the 10-item IMP (Duncan, 2007). The IMP was subsequently expanded to a 31-item instrument, which was proposed to involve five dimensions (Duncan et al., 2009): Listening with Full Attention (LFA), Non-judgmental Acceptance of Self and Child (NJA-SC), Compassion for Self and Child (C-SC), Emotional Awareness of Self and Child (EA-SC), and Self-regulation in Parenting (SRP). Although the IMP has been widely used in research since its development, there are currently no published studies validating this proposed five-factor structure in an English-language population.

A small number of studies have explored the factor structure of translated versions of the IMP. The first such study tested a Dutch translation of the IMP in a Dutch community sample of mothers of 12–15-year-old (*M* = 13.3 years) adolescents (de Bruin et al., 2014). The results did not support Duncan et al.'s proposed 5-factor model. Instead, exploratory and confirmatory factor analyses suggested six factors. The primary difference between de Bruin et al.'s findings and Duncan et al.'s proposed model was that the parent- and child-focussed items relating to compassion, non-judgment and emotional awareness loaded on separate factors, resulting in the six empirically derived dimensions of (1) Listening with Full Attention (LFA), (2) Non-judgmental Acceptance of Parental Functioning (NJAPF), (3) Compassion for the Child (CC), (4) Emotional Awareness of the Child (EAC), (5) Emotional Non-reactivity in Parenting (ENRP), and (6) Emotional Awareness of Self (EAS). In addition, items 3 and 6 were excluded due to low factor loadings, resulting in a 29-item six-factor instrument (de Bruin et al). Another translation of the IMP was tested in a Portuguese-speaking community group of mothers of 1–18-year-olds (*M* = 5.86 years) (Moreira and Canavarro, 2017). Exploratory and confirmatory factor analyses supported the deletion of items 3 and 6, but the findings concerning factor structure were somewhat different from the findings of de Bruin et al. (2014). Listening with Full Attention, Non-judgmental Acceptance of Parental Functioning, Compassion for the Child and Emotional Awareness of the Child contained largely the same items as the Dutch LFA, NJAPF, CC, and EAC factors. However, in this study a new Self-regulation in Parenting (SRP) factor emerged, combining the items from the Dutch ENRP and EAS factors, resulting in a 29-item, five-factor model. Translations of the IMP have also been tested in non-Western countries, including in Hong Kong Chinese parents of 2–19-year-olds (Lo et al., 2018) and Korean parents of 1–18 year-olds (Kim et al., 2018). Numerous items were deleted in both studies, suggesting that the English-language IMP may not easily translate to all other languages or cultures (Lo et al., 2018).

While the differences between the Asian and European studies' findings may be due to linguistic or cultural variations, the differences in the results reported by de Bruin et al. (2014) and Moreira and Canavarro (2017) could partly reflect the differing ages of the children involved in the two studies. Children have different parenting requirements at different developmental stages, such as physical proximity during infancy and autonomy support during adolescence (Karavasilis et al., 2003). It is therefore likely that mindful parenting behaviors differ at different child developmental stages, and separate mindful parenting programs have been offered for parents of infants and children (for example, Potharst et al., 2017). Such differences are not reflected in the current version of the IMP, however. Indeed, some IMP items have limited face validity for parents of pre-verbal children. For example, item 4 (“I listen carefully to my child's ideas, even when I disagree with them”) may only be relevant for parents with children who can express themselves verbally. Therefore, the structure of the IMP should be examined separately in parents of pre-verbal infants and parents of children, to clarify whether the IMP operates equivalently for these two groups of parents.

In addition to child age, the nature of the child's difficulties is important when developing mindful parenting programs. To date, mindful parenting interventions have mainly been studied in parents of children with externalizing problems (for example, Haydicky et al., 2015) or with a range of mental health diagnoses (Emerson et al., 2019). They have not yet been studied in parents of children with only internalizing problems. Both parenting stress and over-reactive parenting have been identified as potential mediators of the relationship between mindful parenting and child externalizing problems (Burgdorf et al., 2019; Emerson et al., 2019). However, little is known about potential mediators between mindful parenting and child internalizing problems. Such mediators may include parental overprotectiveness (Yap et al., 2014), experiential avoidance (Emerson et al., 2019), and beliefs about child anxiety (Francis and Chorpita, 2010). Studies investigating which facets of mindful parenting are most closely related to child internalizing problems and associated parent variables are now needed. Such studies may help guide efforts to develop mindful parenting interventions more specifically targeting child internalizing.

Given the growing research interest in mindful parenting programs, the issues raised above regarding the IMP need to be addressed. The first aim of this study was to examine the fit of the model of mindful parenting proposed by Duncan et al. (2009), as well as the two empirically derived models reported by de Bruin et al. (2014) and Moreira and Canavarro (2017), using confirmatory factor analyses (CFAs). We conducted these analyses separately in parents of infants and parents of children, to explore possible differences in the factor structure of the IMP for these two groups of parents. The second aim of the study was to investigate the relationships between the IMP facets suggested by our CFAs, child internalizing problems, and related parent variables. We hypothesized that more mindful parenting would be related to lower child internalizing problems, as well as lower parenting stress, healthier beliefs and less accommodation regarding child anxiety, and lower parental experiential avoidance. We explored which dimensions of mindful parenting would be most strongly associated with these outcomes.

## Methods

### Participants and Procedure

The study procedures were approved by the relevant institutional Human Research Ethics Committee (approval numbers 183/2019 and 440/2019). A total of 990 participants were recruited from the community, using targeted Facebook advertisements. The advertisement contained a link to the information statement and consent form, hosted on the secure data collection website Qualtrics. People were invited to take part if English was their primary language and they were a parent, or acting in the role of parent, to at least one child aged 0–20 years. There were no exclusion criteria. Participants with more than one child were asked to answer the parenting questions with regard to just one of their children.

From the 990 participants who provided informed consent, 765 participants completed the demographic data and the IMP (Duncan et al., 2009). To increase consistency with de Bruin et al. (2014) and Moreira and Canavarro (2017), we removed the data of fathers (*n* = 41) and the data of parents of children aged 19–20 years of age (*n* = 8), leaving data for the confirmatory factor analyses from 716 mothers (or other female caregivers) of children aged 0–18 years. The age of the mothers or other female caregivers of infants ranged from 22 to 56 years (*M* = 32.25; *SD* = 4.79) and their infants' mean age was 0.90 years (*SD* = 0.78). Mothers or other female caregivers of children were aged between 26 and 58 years (*M* = 39.21, *SD* = 6.60), and the mean age of their children was 8.23 years (*SD* = 4.21). Table 1 contains further information on sample characteristics. A subset (*n* = 245) of these 716 mothers was also asked to complete a set of measures of child internalizing and related parent variables. Questionnaires were presented in random order to reduce order effects. This resulted in a different sample size completing the various questionnaires due to participant drop-out.

**Table 1:** Sample characteristics (*N* = 716).

	**Parents of children**, ***n*** **= 396**		**Parents of infants**, ***n*** **= 320**		**Difference between groups**	
	***n***	**%**	***n***	**%**	**χ^**2**^*(df)***	**^Φ_C_^**
**Child gender**					1.78 (2)	0.05
Male	161	50.3	192	48.6		
Female	201	50.9	159	49.7		
Other	2	0.5				
**Parent relation to child**					0.34 (1)	0.02
Biological mother	386	97.5	314	98.1		
Other female caregiver	10	2.5	6	1.9		
**Caregiver role**					9.57 (2)^**^	0.12
Primary carer	271	68.4	252	78.8		
Equal carer^a^	121	30.6	66	20.6		
Secondary carer	4	1.0	2	0.6		
**No. children in family**					205.16 (3)^***^	0.54
1	75	18.9	228	71.3		
2	198	50.0	70	21.9		
3	100	25.3	14	4.4		
≥4	23	5.8	8	2.5		
**Parent country of residence**					3.00 (1)	0.07
Australia	304	78.6	232	73.0		
Other	83	21.6	86	29.1		
**Parent highest level of education**					0.02 (2)	0.01
Post-graduate or Bachelor degree	290	73.8	236	73.8		
Associate degree or vocational training	53	13.5	44	13.8		
Secondary school or other	50	12.8	40	12.5		
**Parent previous mental health diagnosis**					5.36 (1)^*^	0.09
No	248	62.6	173	54.1		
Yes	148	37.4	147	45.9		
**History of formal mindfulness practice**					4.74 (1)^*^	0.08
Yes	144	36.4	142	44.4		
No	252	63.6	178	55.6		
**Length of mindfulness practice**					2.35 (1)	0.08
<1 year	64	46.0	68	48.9		
≥1 year	75	54.0	71	51.1		
**Frequency of mindfulness practice**					4.85 (1)^*^	0.11
<Monthly	50	36.0	80	57.6		
≥Monthly	89	64.0	59	42.4		

Φ_C_ is Cramer's V effect size, where 0.1–0.3 is a small effect, 0.3–0.5 a moderate effect, and >0.5 a large effect (Cohen, 1988);

a Equal carer is a parent who reports sharing the care of their child approximately equally with another person;

* p ≤ 0.05;

** p ≤ 0.01;

*** *p ≤ 0.001*.

As shown in Table 1, there were several demographic differences between the two groups of mothers. Compared to mothers of children, more mothers of infants identified as a primary carer rather than as an equal carer, and families of infants generally had fewer children. A slightly higher proportion of mothers of infants also reported having previously been diagnosed with a mental health condition and having a history of practicing mindfulness. Amongst mothers who reported a history of mindfulness practice, slightly more mothers of children than infants reported that they currently practiced mindfulness at least monthly.

### Measures

Demographics and Mindfulness Practice Questionnaire: demographic information was collected from participants on the variables presented in Table 1. Participants were also asked whether they had ever engaged in formal mindfulness or other form of meditation or contemplative practice. Response options were one or more of mindfulness, yoga, tai chi, other (participant to specify) or none. Participants who indicated some form of past formal practice were asked to indicate approximately how long they had engaged in that practice. For the purposes of the analyses in this paper, answers were dichotomized into “<*1 year*” and “*1 year or more*.” For those currently practicing, the reported frequency of practice was dichotomized into “*less than monthly*” and “*monthly or more*.” The data reported in this paper relate only to history, length and frequency of formal mindfulness practice.

Interpersonal Mindfulness in Parenting scale (IMP; Duncan, 2007, Duncan et al., 2009): the 31-item IMP measures mindfulness in the parenting context. The items are rated using a 5-point Likert-type scale, where 1 = *Never True*, 2 = *Rarely True*, 3 = *Sometimes True*, 4 = *Often True* and 5 = *Always True*. A total score is calculated by summing the items, with 14 items (1, 5, 9–15, 17, 19, 23, 26, and 29) reverse coded. Higher scores indicate more mindful parenting.

Strengths and Difficulties Questionnaire (SDQ; Goodman, 1997): The SDQ assesses child mental health in children aged 2–18 years. Five subscales relating to emotional problems, peer problems, behavioral problems, hyperactivity, and prosocial behavior are made up of five questions each, with 3-point response scales, where 0 = *Not true*, 1 = *Somewhat true* and 2 = *Certainly true*. In this study, we report only on the Emotional Problems and Peer Problems subscales, combined into an Internalizing Problems scale, where a higher score indicates more problems. The Internalizing Problems scale has good convergent and discriminant validity and internal consistency in general community samples (Goodman et al., 2010).

Depression Anxiety Stress Scales, 21 item version (DASS-21; Lovibond and Lovibond, 1995): the DASS-21 was used to measure parental distress. The DASS-21 is a self-report measure with three scales assessing the emotional states of depression, anxiety and stress. The items are answered on a 4-point Likert-type scale, ranging from 0 (*Did not apply to me at all*) to 3 (*Applied to me very much or most of the time*). Higher scores indicate greater distress. The psychometric properties of the DASS-21 have been reported to be excellent in several studies (e.g., Antony et al., 1998; Crawford and Henry, 2003).

Parental Attitudes, Beliefs and Understanding about Anxiety scale (PABUA; Wolk et al., 2016): the PABUA is a 21-item self-report measure of a parent's beliefs and attitudes about their child's anxiety, consisting of three scales. Overprotection measures parent beliefs about protecting their child from anxiety, with items such as “*It is important that I protect my child from feeling anxious*.” Approach measures beliefs regarding child autonomy and exposure to anxiety, for example “*A way to help my child feel less anxious is to encourage him/her to face his/her fears*.” Finally, Distress measures parent distress in connection with their child's anxiety, for example “*It is hard for me to be with my child when he/she is nervous*.” Items 4, 12, 16, and 21, which form the Approach scale, are reverse scored. The items are answered on a 5-point scale, from 1 = *Strongly disagree* to 5 = *Strongly agree*, with higher scores indicative of less helpful beliefs about anxiety. The PABUA has good convergent and divergent validity, with adequate to good internal consistency (Wolk et al., 2016).

Parental Acceptance and Action Questionnaire (PAAQ; Cheron et al., 2009): the PAAQ is a 15-item self-report measure of experiential avoidance in parenting. Items are rated on a 7-point scale from 1 = *Never true* to 7 = *Always true*, with higher scores indicating more experiential avoidance. Items 1, 5–7, 10, and 11 are reverse scored. The items are summed to create a parental experiential avoidance total score, which measures a parent's unwillingness to witness their child's negative feelings and their inability to manage their own reactions to those negative feelings. Data regarding the PAAQ's concurrent validity and adequate internal consistency have been reported by Cheron et al. (2009).

Parental Accommodation Scale (PAS; Meyer et al., 2018): The 5-item PAS-Behavior scale measures the frequency of parental behaviors aimed at helping their child to lessen or avoid anxiety, with items such as “*I help my child avoid things or perform behaviors so that he or she feels better immediately*.” The items are answered on a 4-point scale ranging from 0 = *Never/almost never* to 3 = *Always/almost always*. Higher scores indicate more unhelpful accommodating behaviors. Meyer et al. (2018) demonstrated the PAS-Behavior scale's convergent validity and good internal consistency.

The parents also completed three other questionnaires that were not included in the current report. The internal consistency (Cronbach's alpha) of the child and parent outcome measures used in this study, other than for the PABUA Approach scale, are reported below in **Table 5**. The PABUA Approach scale was excluded from the analyses due to poor internal consistency (α = 0.28 for mothers of infants, α = 0.41 for mothers of children).

### Statistical Analyses

The confirmatory factor analyses were conducted using AMOS version 25. To check whether the data met the assumption of multivariate normality of distribution underlying structural equation modeling, we screened for multivariate kurtosis and outliers. In both groups of mothers, screening revealed mild multivariate kurtosis and no clear outliers based on an examination of the squared Mahalanobis distance for each case. Goodness-of-fit was assessed against several indices in addition to the chi-square test. Good and adequate fit were indicated, respectively, by normed chi-square (*X*^2^*/df*) ≤ 2 and ≤5, a comparative fit index (CFI) ≥0.95 and ≥0.90, root-mean-square error of approximation (RMSEA) ≤0.05 and ≤0.08, and standardized root mean square residual (SRMR) ≤0.08 and ≤0.10 (Byrne, 2010). We then used SPSS version 26 to conduct a series of simultaneous multiple regression analyses to determine the unique contribution of individual IMP subscales to the prediction of scores on measures of child internalizing and related parent variables.

## Results

### Confirmatory Factor Analysis

We began by testing the fit of the Duncan et al. (2009), de Bruin et al. (2014), and Moreira and Canavarro (2017) models in mothers of children. The fit indices are in Table 2. Based on all the indices used, Duncan et al.'s proposed model (Model C.1) was a poor fit to the data. The factor loadings for items 3 and 6 were low (0.07 and 0.21, respectively) and the loading for item 3 was non-significant. Due to the poor model fit, we did not examine modification indices for this model.

**Table 2:** Fit indices from the confirmatory factor analyses, for mothers of children (*n* = 396).

	**Model**	***X*^**2**^**	***df***	***X*^**2**^*/df***	**CFI**	**RMSEA**	**90% CI for RMSEA**	**SRMR**	**Change from previous model (Δ*X*^2^)**
C.1	Duncan et al. (2009) 31 items	1,698.70^**^	424	4.01	0.750	0.087	[0.083, 0.092]	0.1027	–
C.2	de Bruin et al. (2014) 31 items	944.81^**^	419	2.26	0.897	0.056	[0.052, 0.061]	0.0686	–
C.3	de Bruin et al. (2014) 29 items (excluding items 3 and 6)	764.36^**^	362	2.11	0.919	0.053	[0.048, 0.058]	0.0592	180.45 (57)^*^
C.4	de Bruin et al. (2014) 29 items (covary e18 and e20)	733.53^**^	361	2.03	0.925	0.051	[0.046, 0.056]	0.0598	30.83 (1)^*^
C.5	de Bruin et al. (2014) 29 items (cross-load item 24)	693.41^**^	360	1.93	0.933	0.048	[0.043, 0.054]	0.0575	40.12 (1)^*^
C.6	Moreira and Canavarro (2017) 29 items	835.13^**^	367	2.28	0.906	0.057	[0.052, 0.062]	0.0623	–
C.7	Moreira and Canavarro (2017) 29 items (covary e18 and e20)	808.74^**^	366	2.21	0.911	0.055	[0.050, 0.060]	0.0628	26.39 (1)^*^
C.8	Moreira and Canavarro (2017) 29 items (cross-load item 24)	780.16^**^	365	2.14	0.916	0.054	[0.048, 0.059]	0.0622	28.58 (1)^*^
C.9	Moreira and Canavarro (2017) 29 items (covary e2 and e21)	743.53^**^	364	2.04	0.924	0.051	[0.046, 0.057]	0.0605	36.36 (1)^*^

CFI is Comparative fit index; RMSEA is root-mean-square error of approximation; SRMR is standardized root mean square residual;

* p < 0.01;

** *p < 0.001*.

Next, we examined the fit of the de Bruin et al. model. We began by specifying a six-factor model containing all 31 IMP items (Model C.2), to check whether items 3 and 6 remained problematic. The factor loadings for items 3 (0.08) and 6 (0.04) were again low and non-significant. We therefore excluded those items and specified a 29-item six-factor model (Model C.3). The fit indices ranged from adequate to good, and the fit improved compared to Model C.2. The modification indices for Model C.3 suggested covariance between the errors for two items loading on NJAPF (items 18 and 20). Because both items were related to acceptance of parenting mistakes, we decided to allow these errors to covary (Model C.4). Model fit significantly improved and the fit indices ranged from adequate to good. The modification indices for Model C.4 indicated a cross-loading for item 24, on the CC factor. Item 24 refers to the parent paying close attention to the child when together. As this is similar to several CC items which refer to the parent being attentive to the child in different ways, we made this modification. The revised model (Model C.5) was a reasonably good fit to the data and an improvement on Model C.4. There were no further substantial or theoretically justified error covariances or model misspecifications indicated by the modification indices.

We then tested the 29-item, five-factor Moreira and Canavarro model (Model C.6) in mothers of children. Model C.6 was an adequate to good fit to the data. All factor loadings were significant. The loading for item 10 was 0.36, with all others >0.56. Like the de Bruin et al. model, modification indices suggested an error covariance for items 18 and 20. When this modification was made (Model C.7), the fit improved. The modification indices for Model C.7 suggested the same cross-loading for item 24 on CC. When that cross-loading was allowed, the re-specified model (Model C.8) was again an improvement on the previous model. For Model C.8, modification indices suggested covariance between the errors for items 2 and 21, which both load on the SRP factor. As these items are similar and both relate to pausing before acting, we allowed this error covariance. This resulted in Model C.9, whose indices indicated an adequate to good fit to the data and were a significant improvement on the previous model. No further meaningful modifications were indicated.

In mothers of infants, we followed the same process as set out above. Table 3 contains the fit indices for mothers of infants. The Duncan et al. model (Model I.1) exhibited a poor fit. The factor loadings of items 3 and 6 were low (both 0.03) and non-significant, and the loading for item 10 was low (0.24). We did not check modification indices for this model, due to the poor fit.

**Table 3:** Fit indices from the confirmatory factor analyses, for mothers of infants (*n* = 320).

	**Model**	***X*^**2**^**	***df***	***X*^**2**^*/df***	**CFI**	**RMSEA**	**90% CI for RMSEA**	**SRMR**	**Change from previous model (Δ*X*^2^)**
I.1	Duncan et al. (2009) 31 items	1437.17^**^	424	3.39	0.728	0.087	[0.082, 0.091]	0.0953	–
I.2	de Bruin et al. (2014) 31 items	791.75^**^	419	1.89	0.900	0.053	[0.047, 0.058]	0.0705	–
I.3	de Bruin et al. (2014) 29 items (excluding items 3 and 6)	669.27^**^	362	1.85	0.916	0.052	[0.045, 0.058]	0.0662	122.48 (57)^*^
I.4	de Bruin et al. (2014) 29 items (covary e4 and e28)	649.22^**^	361	1.80	0.921	0.050	[0.044, 0.056]	0.0662	20.05 (1)^*^
I.5	de Bruin et al. (2014) 29 items (covary e4 and e7)	630.76^**^	360	1.75	0.926	0.049	[0.042, 0.055]	0.0660	18.46 (1)^*^
I.6	Moreira and Canavarro (2017) 29 items	705.06^**^	367	1.92	0.907	0.054	[0.048, 0.060]	0.0661	–
I.7	Moreira and Canavarro (2017) 29 items (covary e14 and e29)	666.45^**^	366	1.82	0.918	0.051	[0.045, 0.057]	0.0649	38.61 (1)^*^
I.8	Moreira and Canavarro (2017) 29 items (covary e4 and e28)	645.71^**^	365	1.77	0.923	0.049	[0.043, 0.055]	0.0649	20.74 (1)^*^
I.9	Moreira and Canavarro (2017) 29 items (covary e4 and e7)	626.75^**^	364	1.72	0.928	0.048	[0.041, 0.054]	0.0646	18.96 (1)^*^

CFI is Comparative fit index; RMSEA is root-mean-square error of approximation; SRMR is standardized root mean square residual;

* p < 0.01;

** *p < 0.001*.

We then tested the de Bruin et al. model (Model I.2). The covariance matrix indicated a reasonably good fit to the observed matrix. The loadings for items 3 and 6 were low (both 0.10) and non-significant. The factor loading for item 10 was also low (0.17), but significant (*p* < 0.001). Therefore, items 3 and 6 were excluded and the model re-specified with 29 items (Model I.3). Modification indices suggested error covariances that differed from those found in the sample of mothers of children. For Model I.3, covariance between the errors for CC items 4 and 28, which refer to listening to the child's point of view, was suggested. These errors were allowed to covary, resulting in a significantly improved fit (Model I.4). The modification indices for Model I.4 then suggested covariance between a similar pair of items loading on CC. Items 4 and 7 both relate to allowing a child to express themselves, even in circumstances when this might be difficult for the parent. This modification was made, leading to a further improvement (Model I.5). The modification indices for Model I.5 did not indicate any substantial error covariances or misspecifications to the model.

Last, we examined the the 29-item Moreira and Canavarro model in mothers of infants (Model I.6). Model I.6 was a reasonably good fit. Item 10 had the lowest factor loading (0.28), with all other loadings at least 0.44. All loadings were significant. The modification indices for Model I.6 indicated covariance between the errors for items 14 and 29. As these items both load on the SRP factor and refer to parental over-reactivity to the child when upset, they were allowed to covary. With the model re-specified (Model I.7), the fit improved. Modification indices for Model I.7 then suggested covarying errors for CC items 4 and 28. When this modification was made, the fit improved (Model I.8). For Model I.8, the only substantial change suggested was the covariance of the errors for CC items 4 and 7. With this modification, the fit of the revised model (Model I.9) improved and exhibited a reasonably good fit to the data. No further modifications were warranted.

For both groups of mothers, fewer modifications needed to be made to the de Bruin et al. model to achieve optimum fit. The principal difference between the Moreira and Canavarro and de Bruin et al. models is that the items loading on the Dutch EAS and ENRP factors are combined into the single SRP factor in the Moreira and Canavarro model. Although the Dutch EAS and ENRP factors are closely related, they tap theoretically distinct aspects of parenting, that is emotional self-awareness and non-reactivity. We therefore decided to use the de Bruin et al. model in all following analyses to identify whether these two factors have unique predictive value. The factor loadings for the de Bruin et al. model for mothers of children and infants (Models C.5 and I.5), and the Cronbach's alpha for each scale, are presented in Table 4.

**Table 4:** Standardized factor loadings for 29-item de Bruin et al. (2014) model, for mothers of children (Model C.5) and infants (Model I.5).

		**Mothers of children (*****n*** **= 396)**						**Mothers of infants (*****n*** **= 320)**					
	**Item**	**LFA**	**NJAPF**	**EAC**	**CC**	**EAS**	**ENRP**	**LFA**	**NJAPF**	**EAC**	**CC**	**EAS**	**ENRP**
1	Listening to my child with one ear	0.72						0.65					
9	Rush through activities without being attentive	0.79						0.69					
13	Easily distracted when with my child	0.77						0.72					
19	Not listening, busy thinking about other things	0.78						0.76					
24	Pay close attention to child when together	0.54			0.32			0.72					
15	Hard on myself regarding parenting mistakes		0.70						0.75				
17	Blame myself when times are difficult with child		0.69						0.76				
18	Accept parenting mistakes and move on		0.60						0.63				
20	Give myself a break if I regret my parenting actions		0.55						0.68				
23	Criticize myself for my parenting		0.84						0.76				
26	Think other parents have it easier with parenting		0.64						0.62				
12	Hard to tell what my child is feeling			0.73						0.62			
22	Find it easy to tell when my child is worried			0.74						0.69			
30	Can tell what my child is feeling			0.85						0.77			
4	Listening carefully to child's ideas				0.64						0.37		
7	Allow my child to express their feelings				0.57						0.62		
25	Kind to my child when they upset				0.65						0.67		
27	Nurturing with child when they having a difficult time				0.69						0.74		
28	Try to understand child's point of view				0.71						0.68		
31	Patient with child when they having a hard time				0.70						0.77		
2	Notice how I feel before I take action					0.66						0.65	
8	When upset, I calmly tell child how I feel					0.65						0.49	
16	Try to keep my emotions in balance when upset					0.68						0.72	
21	Pause before reacting, in difficult situations					0.77						0.71	
5	React too quickly to my child						0.71						0.67
10	Difficulty accepting child's growing independence						0.34						0.16
11	Only realize later that feelings affect parenting decisions						0.64						0.68
14	Do things I regret when my child misbehaves						0.77						0.76
29	Get carried away with my feelings when child upsets me						0.76						0.83
	Cronbach's alpha for scale:	0.87	0.84	0.81	0.82	0.78	0.77	0.83	0.85	0.73	0.81	0.73	0.73

*LFA is the Listening with Full Attention scale of the Interpersonal Mindfulness in Parenting questionnaire (IMP); NJAPF is the Non-judgmental Acceptance of Parental Functioning scale of the IMP; EAC is the Emotional Awareness of the Child scale of the IMP; CC is the Compassion for the Child scale of the IMP; EAS is the Emotional Awareness of the Self scale of the IMP; ENRP is the Emotional Non-reactivity in Parenting scale of the IMP*.

### Relationships Between IMP and Demographic and Mindfulness Practice Variables

There were no significant relationships (all *p*s > 0.05) between IMP scores and the background demographic variables, except for small positive associations between IMP scores and parent or child age. These correlations were very small and likely to have no practical significance (e.g., *r* = 0.13, *p* = 0.008 between parent age and IMP score amongst mothers of children). IMP scores were significantly associated with parent mental health for both groups. Mothers of children without a previous mental health diagnosis reported more mindful parenting (*M* = 103.89, *SD* = 12.75) than those with a previous diagnosis (*M* = 98.97, *SD* = 12.75; *t* = −3.72, *p* < 0.001). The same pattern was found amongst mothers of infants, with more mindful parenting in those without a previous diagnosis (*M* = 107.67, *SD* = 12.44), than in those with one (*M* = 104.85, *SD* = 12.43; *t* = −2.02, *p* = 0.044).

IMP scores were also related to some aspects of mindfulness practice. Amongst mothers of children, there was no difference in IMP scores based on history of formal mindfulness practice or the length of that practice history (both *p*s >0.05). However, IMP scores were related to frequency of current practice, with mothers who reported at least monthly practice having higher scores (*M* = 104.92, *SD* = 13.03) than those practicing less than monthly (*M* = 98.28, *SD* = 11.36; *t* = 3.02, *p* = 0.003). In mothers of infants, IMP scores were higher amongst mothers with a history of formal mindfulness practice (*M* = 108.28, *SD* = 12.15), compared to those without that history (*M* = 104.85, *SD* = 12.60; *t* = −2.46, *p* = 0.015), and amongst those who had practiced for more than 1 year (*M* = 111.04, *SD* = 12.37), compared to those who had practiced for less than a year (*M* = 105.71, *SD* = 11.36; *t* = −2.65, *p* = 0.009). However, IMP scores did not differ according to frequency of current practice (*p* > 0.05) in this group.

### Relationships Between IMP and Child and Parent Outcome Variables

Correlations between demographic and mindfulness practice variables, and child and parent outcome variables, were calculated to determine whether any of these variables should be included as control variables in the regression analyses. These correlations are shown in Table 5. Demographic or mindfulness practice variables were included as control variables if the correlations between those variables and the child or parent outcome variables were significant, or where the correlation coefficient was 0.25 or more. We included control variables based on the size of the correlation coefficient as well as statistical significance because of the smaller sample size of mothers of infants.

**Table 5:** Correlations between IMP subscales, demographic and mindfulness practice variables, and outcome variables, for mothers of children and infants.

**Predictors**	**Mothers of children aged 3–18 years^a^**						**Mothers of infants aged 0–2 years**				
	**SDQ** **Internalizing**	**DASS** **Stress**	**PABUA** **Over–protection**	**PABUA** **Distress**	**PAAQ** **Total**	**PAS** **Behavior**	**DASS** **Stress**	**PABUA** **Over–protection**	**PABUA** **Distress**	**PAAQ** **Total**	**PAS** **Behavior**
	α = 0.70	α = 0.85	α = 0.86	α = 0.71	α = 0.83	α = 0.77	α = 0.87	α = 0.88	α = 0.57	α = 0.81	α = 0.78
	−0.87^b^									
LFA	−0.21^**^	−0.29^***^	−0.14	−0.35^***^	−0.39^***^	−0.31^***^	−0.26^*^	0.03	−0.30^*^	−0.25	−0.06
CC	−0.17^*^	−0.15^*^	−0.04	−0.45^***^	−0.47^***^	−0.12	−0.12	−0.06	−0.35^**^	−0.53^***^	−0.02
NJAPF	−0.40^***^	−0.50^***^	−0.34^***^	−0.48^***^	−0.69^***^	−0.44^***^	−0.53^***^	−0.20	−0.38^**^	−0.65^***^	−0.29^*^
EAC	−0.29^***^	−0.14	−0.01	−0.39^***^	−0.30^***^	−0.18^*^	0.02	−0.02	−0.19	−0.24	−0.02
ENRP	−0.32^***^	−0.40^***^	−0.16	−0.46^***^	−0.58^***^	−0.26^**^	−0.36^***^	−0.13	−0.52^***^	−0.59^***^	−0.35^**^
EAS	−0.28^***^	−0.24^**^	−0.09	−0.38^***^	−0.45^***^	−0.15	−0.28^*^	−0.13	−0.37^**^	−0.57^***^	−0.31^*^
Parent age	−0.01	−0.29^***^	−0.14	−0.08	−0.09	−0.20^*^	−0.18	0.02	−0.06	−0.17	−0.13
Child age	0.24^**^	−0.10	−0.10	0.00	0.02	−0.07	−0.06	−0.06	−0.15	−0.35^**^	−0.09
Child gender^c^	0.12	0.07	0.14	0.14	0.11	0.22^**^	−0.10	0.06	−0.06	−0.08	0.03
Mental health^d^	0.24^*^	0.26^***^	0.16^*^	0.11	0.27^***^	0.27^***^	0.30^**^	0.07	0.00	0.05	0.12
History of practice^e^	0.07	0.09	−0.08	−0.15	−0.03	0.07	0.00	−0.09	−0.23	−0.13	0.02
Length of practice^f^	−0.14	−0.06	0.08	−0.20	−0.15	0.01	−0.24	−0.13	−0.15	−0.24	−0.30
Frequency of practice^g^	0.03	−0.08	0.01	−0.16	−0.09	−0.17	−0.24	−0.03	−0.02	0.24	0.02

a For SDQ Internalizing, this group comprises mothers of children aged 2–18 years (as SDQ data not available for infants under 2 years);

b Cronbach's alpha is reported separately for the different age categories of SDQ, that is, 0.70 (2–4 years), 0.71 (5–10 years), and 0.87 (11–17 years). No alpha could be calculated for the SDQ (18 years) as there was only 1 mother of a child aged 18 years;

c 0 = females and 1 = males;

d 0 = no previous mental health diagnosis and 1 = previous mental health diagnosis;

e 0 = no history of mindfulness practice and 1 = some history of mindfulness practice;

f 0 = <1 year history of mindfulness practice and 1 = one or more years history of mindfulness practice;

g 0 = currently practicing less than monthly and 1 = currently practicing monthly or more; SDQ Internalizing is the Internalizing scale of the Strengths and Difficulties Questionnaire; DASS Stress is the Stress scale of the Depression Anxiety Stress Scales; PABUA Overprotection is the Overprotection scale of the Parental Attitudes, Beliefs, and Understanding of Anxiety scale; PABUA Distress is the Distress scale of the Parental Attitudes, Beliefs, and Understanding of Anxiety scale; PAAQ Total is the Total scale from the Parental Acceptance and Action Questionnaire; PAS Behavior is the Behavior scale of the Parental Accommodation Scale; LFA is the Listening with Full Attention scale of the Interpersonal Mindfulness in Parenting questionnaire (IMP); CC is the Compassion for the Child scale of the IMP; NJAPF is the Non-judgmental Acceptance of Parental Functioning scale of the IMP; EAC is the Emotional Awareness of the Child scale of the IMP; ENRP is the Emotional Non-reactivity in Parenting scale of the IMP; EAS is the Emotional Awareness of the Self scale of the IMP;

* p ≤ 0.05;

** p ≤ 0.01;

*** *p ≤ 0.001*.

Tables 6, 7 detail the results of the regression analyses for child internalizing and the parent outcome variables. Child internalizing problems (for children aged 2–18) were uniquely predicted by the NJAPF and EAC facets, when all other variables were held constant in the equation. For mothers of children, all parent outcomes had a unique association with NJAPF. Parent distress regarding child anxiety was also predicted by EAC and CC, and parental experiential avoidance was also predicted by CC. A different pattern was found for mothers of infants. Parent stress was uniquely predicted by NJAPF, parent distress regarding child anxiety was predicted by ENRP, experiential avoidance by NJAPF and EAS, and accommodation of child anxiety by EAS and CC.

**Table 6:** Regression analysis of demographic and mindful parenting scale predictors of child internalizing problems (SDQ Internalizing), for mothers of children aged 2–18 years (*n* = 163).

	**Model 1**				**Model 2**			
	***R*^**2**^**	**β**	***t***	***sr^**2**^***	***R*^**2**^**	**β**	***t***	***sr^**2**^***
	0.09^***^				0.26^***^			
Child age		0.25^***^	3.27	0.06		0.21^**^	3.01	0.04
Mental health^a^		0.19^**^	2.54	0.04		0.10	1.32	0.01
LFA						0.01	0.10	0.00
CC						0.10	1.06	0.01
EAC						−0.18^*^	−2.20	0.02
NJAPF						−0.30^***^	−3.24	0.05
ENRP						−0.06	−0.48	0.00
EAS						−0.08	−0.77	0.00

a 0 = no previous mental health diagnosis and 1 = previous mental health diagnosis; LFA is the Listening with Full Attention scale of the Interpersonal Mindfulness in Parenting questionnaire (IMP); CC is the Compassion for the Child scale of the IMP; EAC is the Emotional Awareness of the Child scale of the IMP; NJAPF is the Non-judgmental Acceptance of Parental Functioning scale of the IMP; ENRP is the Emotional Non-reactivity in Parenting scale of the IMP; EAS is the Emotional Awareness of the Self scale of the IMP;

* p ≤ 0.05;

** p ≤ 0.01;

*** *p ≤ 0.001*.

**Table 7:** Regression analyses of mindful parenting scale predictors of parent outcome variables, for mothers of infants and children.

	**Mothers of children aged 3–18 years**								**Mothers of infants aged 0–2 years**							
	**Model 1**				**Model 2**				**Model 1**				**Model 2**			
	***R*^**2**^**	**β**	***t***	***sr^**2**^***	***R*^**2**^**	**β**	***t***	***sr^**2**^***	***R*^**2**^**	**β**	***t***	***sr^**2**^***	***R*^**2**^**	**β**	***t***	***sr^**2**^***
**DASS Stress**	***n*** **= 167**								***n*** **= 75**							
Predictors:	0.13^***^				0.32^***^				0.09^**^				0.38^***^			
Parent age		−0.26^***^	−3.56	0.07		−0.19^**^	−2.69	0.03		–	–	–		–	–	–
Mental health^a^		0.22^**^	2.94	0.05		0.11	1.63	0.01		0.30^**^	2.67	0.09		0.20^*^	2.04	0.04
LFA						−0.06	−0.69	0.00						−0.14	−1.21	0.01
CC						0.10	1.12	0.01						0.15	1.12	0.01
EAC						−0.04	−0.58	0.00						0.15	1.36	0.02
NJAPF						−0.30^***^	−3.36	0.05						−0.41^***^	−3.55	0.12
ENRP						−0.20	−1.85	0.01						−0.11	−0.78	0.01
EAS						0.00	−0.01	0.00						−0.16	−1.13	0.01
**PABUA Overprotection**	***n*** **= 156**								***n*** **= 66**							
Predictors:	0.03^*^				0.13^**^				0.03^*^							
Mental health^a^		0.16^*^	1.97	0.02		0.05	0.65	0.00		–	–	–				
LFA						−0.07	−0.68	0.00		0.14	0.92	0.01				
CC						0.05	0.51	0.00		0.04	0.19	0.00				
EAC						0.05	0.55	0.00		0.00	0.01	0.00				
NJAPF						−0.36^***^	−3.56	0.07		−0.19	−1.24	0.02				
ENRP						0.06	0.48	0.00		−0.05	−0.25	0.00				
EAS						0.01	0.08	0.00		−0.11	−0.61	0.01				
**PABUA Distress**	***n*** **= 156**								***n*** **= 66**							
Predictors:	0.36^***^								0.29^**^							
LFA		0.00	−0.03	0.00						−0.05	−0.37	0.00				
CC		−0.21^*^	−2.37	0.02						−0.09	−0.56	0.00				
EAC		−0.20^**^	−2.68	0.03						0.04	0.31	0.00				
NJAPF		−0.31^***^	−3.68	0.06						−0.14	−1.05	0.01				
ENRP		−0.10	0.98	0.00						−0.37^*^	−2.29	0.06				
EAS		0.01	0.05	0.00						−0.04	−0.22	0.00				
**PAAQ Total**	***n*** **= 148**								***n*** **= 64**							
Predictors:	0.07^***^				0.57^***^				0.12^**^				0.67^***^			
Child age										−0.35^***^	−2.90	0.12		−0.27^***^	−3.40	0.07
Mental health^a^		0.27^***^	3.33	0.07		0.09	1.61	0.01					–	–	–
LFA						0.03	0.44	0.00						0.17	1.90	0.02
CC						−0.22^**^	−2.91	0.03						−0.21	−1.90	0.02
EAC						−0.06	−0.87	0.00						0.07	0.79	0.00
NJAPF						−0.50^***^	−6.71	0.14						−0.41^***^	−4.45	0.12
ENRP						−0.15	−1.62	0.01						−0.21	−1.89	0.02
EAS						−0.01	−0.12	0.00						−0.24^*^	−2.11	0.03
**PAS Behavior**	***n*** **= 143**								***n*** **= 59**							
Predictors:	0.13^***^				0.28^***^				0.03				0.27^*^			
Parent age		−0.15	−1.88	0.02		−0.07	−0.94	0.00		–	–	–		–	–	–
Child gender^b^		0.19^*^	2.32	0.03		0.15^*^	2.03	0.02		–	–	–		–	–	–
Mental health^a^		0.23^**^	2.87	0.05		0.12	1.58	0.01		–	–	–		–	–	–
Length of practice^c^																
<1 year		–	–	–		–	–	–		0.09	0.62	0.01		0.12	0.84	0.01
≥1 year		–	–	–		–	–	–		−0.12	−0.82	0.01		−0.02	−0.11	0.00
LFA						−0.18	−1.85	0.02						0.10	0.72	0.01
CC						0.12	1.14	0.01						0.35^*^	2.02	0.06
EAC						−0.10	−1.24	0.01						0.06	0.39	0.00
NJAPF						−0.35^***^	−3.50	0.07						−0.11	−0.79	0.01
ENRP						0.03	0.26	0.00						−0.34	−1.90	0.05
EAS						0.03	0.25	0.00						−0.35^*^	−1.97	0.06

a 0 = no previous mental health diagnosis and 1 = previous mental health diagnosis;

b 0 = females and 1 = males;

c 0 = <1 year history of mindfulness practice and 1 = one or more years history of mindfulness practice; DASS Stress is the Stress scale of the Depression Anxiety Stress Scales; PABUA Overprotection is the Overprotection scale of the Parental Attitudes, Beliefs, and Understanding of Anxiety scale; PABUA Distress is the Distress scale of the Parental Attitudes, Beliefs, and Understanding of Anxiety scale; PAAQ Total is the Total scale from the Parental Acceptance and Action Questionnaire; PAS Behavior is the Behavior scale of the Parental Accommodation Scale; LFA is the Listening with Full Attention scale of the Interpersonal Mindfulness in Parenting questionnaire (IMP); CC is the Compassion for the Child scale of the IMP; EAC is the Emotional Awareness of the Child scale of the IMP; NJAPF is the Non-judgmental Acceptance of Parental Functioning scale of the IMP; ENRP is the Emotional Non-reactivity in Parenting scale of the IMP; EAS is the Emotional Awareness of the Self scale of the IMP;

* p ≤ 0.05;

** p ≤ 0.01;

*** *p ≤ 0.001*.

## Discussion

### The Structure of Mindful Parenting

This study sought to examine the structure of mindful parenting, to determine whether it differed for parents of infants and parents of children, and to investigate the relationships between the facets of mindful parenting, child internalizing, and parent variables related to child internalizing. In relation to factor structure, the model proposed by Duncan et al. (2009) was a poor fit in both groups of mothers. In contrast, the de Bruin et al. (2014) and Moreira and Canavarro (2017) models were an adequate to good fit in both mothers of children and infants. Amongst mothers of children, the slightly better fit indices and lower number of modifications required suggested the de Bruin et al. model was a marginally better fit to the data. Amongst mothers of infants, the indices showed both models to be a reasonably good fit, although the de Bruin et al. model again required fewer modifications to achieve best fit. The divergence of fit between the proposed Duncan et al. model on the one hand, and the de Bruin et al. and Moreira and Canavarro models on the other, supports the separation of the parent- and child-focused items relating to compassion, non-judgment, and emotional awareness onto separate factors. This separation of parent- and child-focused items in an English-speaking group of mothers confirms that this is a reflection of the construct of mindful parenting rather than an artifact of the translation process or a reflection of cultural differences. Our results also confirm that items 3 and 6 should be deleted from the IMP, as suggested by de Bruin et al. (2014) and Moreira and Canavarro (2017).

The fit of the de Bruin et al. (2014) and Moreira and Canavarro (2017) models in both groups of mothers also shows that the construct of mindful parenting is similar for mothers of children and mothers of infants. One potential issue regarding the operation of the IMP in parents of pre-verbal infants was that some items appeared to have limited face validity. For example, the wording of items 4 (“I listen carefully to my child's ideas, even when I disagree with them”) and 28 (“I try to understand my child's point of view, even when his/her opinions do not make sense to me”) appears relevant only to parents of children who can verbally express ideas or opinions. For item 28, the loadings were very similar across mothers of children (0.71) and infants (0.68). For item 4, although the loading for mothers of infants (0.37) was lower than for mothers of children (0.64), it was significant. In addition, amongst mothers of infants but not children, the errors for items 4 and 28 were correlated. This pattern of factor loadings, and the error covariance for mothers of infants only, suggests that even though infants do not have sufficient verbal skills to express their opinions, these items are measuring an underlying understanding by mothers that infants can communicate in other ways, such as through displays of emotion. Mothers therefore appear to interpret these items in a manner that is applicable to the developmental age of their child.

There was also some variation between the two groups of mothers in the size of the loadings for item 10 (“I have difficulty accepting my child's growing independence”). This item had a loading on the ENRP facet of only 0.16 for mothers of infants, and only 0.34 for mothers of children. As the group of mothers of children had a broader range of children, including adolescents in the process of gaining independence from their parents (Moretti and Peled, 2004), it is expected that item 10 would be more relevant to those mothers. However, both loadings were still low, raising the question as to whether it is a good indicator of non-reactivity. This item was also problematic in the unpublished validation of the 10-item IMP (Duncan, 2007), where it showed low correlations with other items. Further investigations could help clarify whether item 10 should be retained in the IMP.

### Relationship Between Mindful Parenting, Child Internalizing, and Parent Outcome Variables

The regression analyses conducted in this study show that several facets of mindful parenting predict child internalizing problems and related parent outcomes, after controlling for demographic and mindfulness practice variables. Child internalizing problems were predicted by the NJAPF and EAC facets, when all other variables in the equation were held constant. Children have less internalizing problems if their mothers are less judgmental about their own parental functioning. Previously, adolescents have been found to be less anxious and depressed if their parents are less judgmental about themselves as parents (Geurtzen et al., 2015), so the present results confirm this relationship in mothers of a wider age range of children. Mothers with greater emotional awareness regarding their child also had children with less internalizing problems. From the child's perspective, having emotionally competent parents facilitates adaptive processing of emotional experience (Morris et al., 2017). There are various ways in which being more accepting of one's own parental functioning and more emotionally aware could result in children with less internalizing problems. Emotionally competent parents model helpful emotion regulation strategies, including acceptance, thereby providing opportunities for their children to learn these behaviors (Morris et al., 2017). In turn, children with better emotion regulation skills have fewer internalizing problems (Suveg et al., 2011). However, the cross-sectional nature of the data means that alternative explanations are possible. For example, having an anxious child who avoids certain activities like engaging in sports or interacting with other children at school or in social settings may cause a parent to negatively judge their abilities as a parent. Finally, it is also possible that being more judgmental regarding one's own parental functioning or less emotionally aware regarding one's child indicate an underlying predisposition to anxiety, such as negative affect (Barlow, 2000), which predicts child internalizing (Drake and Ginsburg, 2012).

Parent stress was predicted by NJAPF in both mothers of children and infants. Mothers are less stressed if they are less judgmental regarding their own functioning as a parent. These results are consistent with an earlier study by Moreira and Canavarro (2018), who found that non-judgmental acceptance mediates the relationship between self-critical rumination and parenting stress. It seems likely that parents who judge their own performance as a parent less harshly would have lower levels of general stress because they would be less likely to try to meet overly high standards of parenting and be less punishing of themselves for perceived failures to meet those standards (Moreira and Canavarro, 2018).

Parent beliefs and attitudes about child anxiety were predicted by NJAPF, EAC, and CC in mothers of children, but only by ENRP in mothers of infants. Specifically, mothers of children are less likely to believe they need to protect their child from anxiety and are less distressed by their child's anxiety, if they are less judgmental regarding their own functioning as a parent and more emotionally aware and compassionate regarding their child. Parents who find it difficult to understand their child's emotions, including anxiety, may experience distress because they lack skills to manage their child's or their own reactions to that emotional state (Izard et al., 2011). This may also reflect an understanding that anxiety is a normal emotion that everyone will experience at times and, as such, is not something that parents need to guard against in their children. In contrast, mothers of infants experienced less distress regarding child anxiety if they were less emotionally reactive in their parenting. Emotional self-regulation may be important in helping parents of infants to cope with any distress associated with their infant, because the limited capacity of infants to regulate themselves means they must rely on parents' regulatory abilities (Rutherford et al., 2015).

Parental experiential avoidance was predicted by NJAPF and CC in mothers of children and NJAPF and EAS in mothers of infants. Mothers of children are less avoidant if they are less judgmental regarding their parenting and more compassionate with their child. Experientially avoidant parents have difficulty experiencing their own thoughts and emotions in relation to their child's negative emotions (Cheron et al., 2009). More compassionate parents of children may be less avoidant because they are more actively focused upon supporting their child than on their own psychological discomfort. Alternatively, parents who are less avoidant could find it easier to be compassionate toward their child because they are not using attentional resources to manage their own internal state (Kashdan et al., 2008). Mothers of infants are less avoidant if they are less judgmental regarding their parenting and more emotionally self-aware. It is interesting that emotional self-awareness is only predictive of parental experiential avoidance in mothers of infants, and not children. As noted above, infants are less able than older children to regulate themselves and are therefore more likely to be dysregulated for reasons that may not be obvious, which could be frustrating or distressing to a parent. It is possible that parents who are more emotionally self-aware and regulated will be more likely to realize that the psychological discomfort they experience in such situations is a normal emotional reaction to parenting an infant and that this psychological discomfort need not be avoided or suppressed.

Last, parental accommodation of child anxiety was predicted by NJAPF in mothers of children and by EAS and CC in mothers of infants. Mothers of children are less accommodating of their child's anxiety if they are less judgmental regarding their own parenting, whereas mothers of infants are less accommodating if they are more emotionally self-aware and less compassionate with their infant. Compassion involves engaging with someone's suffering rather than avoiding it (Carona et al., 2017), for example through accommodation or overprotection. The finding that *lower* compassion predicts less accommodation behavior therefore seems contradictory to this view of compassion. However, this finding is consistent with the evolutionary perspective that the purpose of a mother-infant attachment relationship is to provide physical and emotional comfort to the infant (Paquette, 2004). While parental overprotectiveness is generally seen as a risk factor for child anxiety (Yap et al., 2014), this is not the case for infants (Möller et al., 2015).

Conducting separate regression analyses for mothers of children and infants has disclosed a different pattern of findings regarding the most important predictors for each group of mothers. For mothers of children, non-judgmental acceptance of parental functioning predicted all parent outcomes related to child internalizing problems and was in each case the largest predictor, making it the most important predictor of outcomes for this group of mothers. This facet might be relevant in this group of mothers because they interpret their child's behavior as reflecting upon the adequacy of their parenting. However, for mothers of infants only, the two facets relating to self-awareness and self-regulation, EAS and ENRP, appear to be important. This is likely to be related to the developmental stage of infants compared to children. The relative inability of all infants to self-regulate requires mothers of infants to assist their infants by regulating *themselves* emotionally and behaviourally. Mothers of infants may be less likely to interpret their infant's behavior as related to the adequacy of their parenting, perhaps because there is a general understanding that infants, unlike children, cannot regulate their own behavior. Our finding regarding the importance of EAS is also consistent with a recent study that investigated the relationship between self-reported mindful parenting, and the quality of interactions between mothers and their 0–4 year-old child (Potharst et al., 2020). In that study, higher EAS predicted higher quality interactions between mother and child. It was suggested that mothers' emotional self-awareness is an underlying requirement for conscious decision-making in parenting and therefore affects behaviors toward the child (Potharst et al., 2020).

### Clinical Implications

The findings discussed above have potentially important clinical implications. First, in line with evidence that mindful parenting and general trait mindfulness are correlated (Meppelink et al., 2016), the present results showed mindful parenting was related to formal mindfulness practice. However, these relationships were weak, indicating that a parent's general mindfulness practice may not have a meaningful impact on their ability to be mindful with their child. Further, as increases in mindful parenting, but not general mindfulness, predict reductions in child psychopathology (Meppelink et al., 2016), families managing child psychopathology may benefit more from mindful parenting programs targeted specifically toward parenting difficulties, rather than from general mindfulness programs.

Second, mindful parenting interventions may be useful in treating child internalizing problems. While cognitive-behavioral therapy (CBT) enjoys the most empirical support as a treatment for child anxiety disorders (MacPherson and Fristad, 2014), a remission rate of 59% across these disorders (James et al., 2013) shows the clear need for additional treatment approaches that cater to those families not helped by CBT. Parent psychopathology and underlying emotion regulation deficits (Aldao et al., 2010) are barriers to the effective treatment of child psychopathology (Maliken and Katz, 2013). Addressing these parental difficulties, for example through a mindful parenting program, is therefore likely to improve child outcomes.

Finally, there is a need to consider the focus of mindful parenting interventions offered to families both in terms of the child's age and the nature of a child's difficulties. In relation to child age, the present results showed a different pattern of predictors for mothers of children vs. infants, suggesting that parents might benefit more from attending programs that are tailored to target the most relevant facets of mindful parenting for parents with children in the relevant age group. Regarding the nature of the child's difficulties, mindful parenting interventions have, to date, largely been targeted to parents of children with externalizing problems, who tend to experience greater reactivity toward their children as a result of elevated parenting stress (Bögels et al., 2010). However, the ENRP facet of mindful parenting did not predict the majority of outcome variables in this study. Instead, NJAPF, CC, EAC, and EAS predicted child internalizing and related parent variables. Accordingly, in mindful parenting interventions for families with internalizing children, it may be important to focus on building non-judgment, compassion and emotional awareness in parents, rather than targeting non-reactivity. At the time of this study, we are not aware of any published research regarding the effectiveness of mindful parenting interventions specifically aimed at families of children with internalizing problems.

### Limitations

There are limitations to note in connection with this study. First, as the IMP validation was undertaken only with mothers, the results are not generalizable to fathers. We are unaware of any investigations of the IMP's factor structure in father-only samples, so a gap remains in our understanding of how the construct of mindful parenting may compare in fathers and mothers. This issue is an important one to address because it informs the question of whether mindful parenting programs, which are currently the same for mothers and fathers, should be tailored to reflect any gender differences in mindful parenting. Second, we only considered the structure of mindful parenting in infants aged 0–2 years and children aged 3–18 years. The group of children in particular had a broad age range, and given that parenting children at each end of this age range may be quite different, it would be interesting for future studies to look at mindful parenting in more precise age groups. Lastly, although we have identified several parent variables that might mediate the relationship between mindful parenting and child internalizing problems, including parental experiential avoidance, beliefs about child anxiety and overprotectiveness, our data are cross-sectional so no meaningful path analyses could be conducted. Since no conclusions can be drawn about the directions of effect from the present results, future studies with longitudinal data are now needed to test these potential mediators.

## Conclusion

This study shows for the first time that the IMP is a valid measure of mindful parenting in English-speaking, community-recruited mothers. Importantly, it also confirms that the IMP operates similarly amongst mothers of pre-verbal infants and mothers of children. Mindful parenting, in particular the facets relating to non-judgmental acceptance of parenting, compassion and emotional awareness, predicts child internalizing problems and parent variables related to child internalizing problems. Mindful parenting programs have the potential to help the substantial proportion of families of children with internalizing problems who are not currently well-served by CBT, including those families grappling with parental psychopathology or emotion regulation difficulties.

## Data Availability Statement

The raw data supporting the conclusions of this article will be made available by the authors, without undue reservation.

## Ethics Statement

The studies involving human participants were reviewed and approved by University of Sydney Human Research Ethics Committee. The patients/participants provided their written informed consent to participate in this study.

## Author Contributions

VB designed the study, collected, and analyzed the data, wrote the first version of the manuscript and revised subsequent versions. MS reviewed and revised the design, statistical analyses, and each version of the manuscript. All authors contributed to the article and approved the submitted version.

## Conflict of Interest

The authors declare that the research was conducted in the absence of any commercial or financial relationships that could be construed as a potential conflict of interest.

## References

[B1] Aldao A. Nolen-Hoeksema S. Schweizer S. (2010). Emotion-regulation strategies across psychopathology: a meta-analytic review. Clin. Psychol. Rev. 30, 217–237. 10.1016/j.cpr.2009.11.004 20015584

[B2] Antony M. M. Bieling P. J. Cox B. J. Enns M. W. Swinson R. P. (1998). Psychometric properties of the 42-item and 21-item versions of the depression anxiety stress scales (DASS) in clinical groups and a community sample. Psychol. Assess. 10, 176–181. 10.1037/1040-3590.10.2.176

[B3] Barlow D. H. (2000). Unravelling the mysteries of anxiety and its disorders from the perspective of emotion theory. Am. Psychol. 55, 1247–1263. 10.1037/0003-066X.55.11.1247 11280938

[B4] Bögels S. M. Lehtonen A. Restifo K. (2010). Mindful parenting in mental health care. Mindfulness 1, 107–120. 10.1007/s12671-010-0014-5 21125026 PMC2987569

[B5] Burgdorf V. Szabó M. Abbott M. J. (2019). The effect of mindfulness interventions for parents on parenting stress and youth psychological outcomes: a systematic review and meta-analysis. Front. Psychol. 10:1336. 10.3389/fpsyg.2019.01336 31244732 PMC6562566

[B6] Byrne B. M. (2010). Structural Equation Modelling With AMOS, 2nd edn. Ottawa, ON: Routledge.

[B7] Carona C. Rijo D. Salvador C. Castilho P. Gilbert P. (2017). Compassion-focused therapy with children and adolescents. Br. J. Psychol. Adv. 23, 240–252. 10.1192/apt.bp.115.015420

[B8] Cheron D. M. Ehrenreich J. T. Pincus D. B. (2009). Assessment of parental experiential avoidance in a clinical sample of children with anxiety disorders. Child Psychiatry Hum. Dev. 40, 383–403. 10.1007/s10578-009-0135-z 19280337

[B9] Cohen J. (1988). Statistical Power Analysis for the Behavioural Sciences. Routledge.

[B10] Crawford J. R. Henry J. D. (2003). The Depression Anxiety Stress Scales (DASS): normative data and latent structure in a large non-clinical sample. Brit. J. Clin. Psychol. 42, 111–131. 10.1348/014466503321903544 12828802

[B11] de Bruin E. I. Zijlstra B. J. H. Geurtzen N. van Zundert R. M. P. van de Weijer-Bergsma E. Hartman E. E.. (2014). Mindful parenting assessed further: psychometric properties of the Dutch version of the Interpersonal Mindfulness in Parenting scale (IMP). Mindfulness 5, 200–212. 10.1007/s12671-012-0168-4 25126133 PMC4130420

[B12] Drake K. L. Ginsburg G. S. (2012). Family factors in the development, treatment, and prevention of childhood anxiety disorders. Clin. Child Fam. Psychol. Rev. 15, 144–162. 10.1007/s10567-011-0109-0 22241071

[B13] Duncan L. G. (2007). Assessment of Mindful Parenting Among Parents of Early Adolescents: Development and Validation of the Interpersonal Mindfulness in Parenting Scale. Doctoral Dissertation, Pennsylvania State University.

[B14] Duncan L. G. Coatsworth J. Greenberg M. T. (2009). A model of mindful parenting: Implications for parent-child relationships and prevention research. Clin. Child Fam. Psychol. Rev. 12, 255–270. 10.1007/s10567-009-0046-3 19412664 PMC2730447

[B15] Emerson L. M. Aktar E. de Bruin E. Potharst E. Bögels S. (2019). Mindful parenting in secondary child mental health: key parenting predictors of treatment effects. Mindfulness. 10.1007/s12671-019-01176-w

[B16] Francis S. E. Chorpita B. F. (2010). Development and evaluation of the parental beliefs about anxiety questionnaire. J. Psychopathol. Behav. Assess. 32, 138–149. 10.1007/s10862-009-9133-5

[B17] Geurtzen N. Scholte R. H. J. Engels R. C. M. E. Tak Y. R. van Zundert R. M. P. (2015). Association between mindful parenting and adolescents' internalising problems: non-judgmental acceptance of parenting as core element. J. Child Fam. Stud. 24, 1117–1128. 10.1007/s10826-014-9920-9

[B18] Goodman A. Ploubidis D. L. Lamping G. B. (2010). When to use broader internalising and externalising subscales instead of the hypothesised five subscales on the Strengths and Difficulties Questionnaire (SDQ): data from British parents, teachers and children. J. Abnorm. Child Psychol. 38, 1179–1191. 10.1007/s10802-010-9434-x 20623175

[B19] Goodman R. (1997). The strengths and difficulties questionnaire: a research note. J. Child Psychol. Psychiatry 38, 581–586. 10.1111/j.1469-7610.1997.tb01545.x 9255702

[B20] Haydicky J. Shecter C. Wiener J. Ducharme J. M. (2015). Evaluation of MBCT for adolescents with ADHD and their parents: impact on individual and family functioning. J. Child Fam. Stud. 24, 76–94. 10.1007/s10826-013-9815-1

[B21] Izard C. E. Woodburn E. M. Finlon K. J. Krauthamer-Ewing E. S. Grossman S. R. Seidenfeld A. (2011). Emotion knowledge, emotion utilization, and emotion regulation. Emot. Rev. 3, 44–52. 10.1177/175407391038097219956781

[B22] James A. C. James G. Cowdrey F. A. Soler A. Choke A. (2013). Cognitive behavioural therapy for anxiety disorders in children and adolescents. Cochrane Database Syst. Rev. CD004690. 10.1002/14651858.CD004690.pub3 23733328

[B23] Kabat-Zinn J. Kabat-Zinn M. (1997). Everyday Blessings: The Inner Work of Mindful Parenting. New York, NY: First Trade.

[B24] Karavasilis L. Doyle A. B. Markiewicz D. (2003). Associations between parenting style and attachment to mother in middle childhood and adolescence. Int. J. Behav. Dev. 27, 153–164. 10.1080/0165025024400015

[B25] Kashdan T. B. Zvolensky M. J. McLeish A. C. (2008). Anxiety sensitivity and affect regulatory strategies: individual and interactive risk factors for anxiety-related symptoms. J. Anxiety Disord. 22, 429–440. 10.1016/j.janxdis.2007.03.011 17449221 PMC2673808

[B26] Kim E. Krägeloh C. U. Medvedev O. N. Duncan L. G. Singh N. N. (2018). Interpersonal mindfulness in parenting scale: testing the psychometric properties of a Korean version. Mindfulness 10, 516–528. 10.1007/s12671-017-0871-2

[B27] Lo H. H. M. Leung J. W. K. Duncan L. G. Ma Y. Siu A. F. Y. Chan S. K. C. (2018). Validating of the interpersonal mindfulness in parenting scale in Hong Kong Chinese. Mindfulness 9, 1390–1401. 10.1007/s12671-017-0879-7

[B28] Lovibond P. F. Lovibond S. H. (1995). The structure of negative emotional states: comparison of the depression anxiety stress scales (dass) with the beck depression and anxiety inventories. Behav. Res. Therapy 33, 335–343. 10.1016/0005-7967(94)00075-U 7726811

[B29] MacPherson H. A. Fristad M. A. (2014). Evidence-based psychosocial treatments for pediatric mood and anxiety disorders. Curr. Treat. Opt. Psychiatry 1, 48–65. 10.1007/s40501-013-0002-1

[B30] Maliken A. C. Katz L. F. (2013). Exploring the impact of parental psychopathology and emotion regulation on evidence-based parenting interventions: a transdiagnostic approach to improving treatment effectiveness. Clin. Child Fam. Psychol. Rev. 16, 173–186. 10.1007/s10567-013-0132-4 23595362

[B31] Meppelink R. de Bruin E. I. Wanders-Mulder F. H. Vennik C. J. Bögels S. M. (2016). Mindful parenting training in child psychiatric settings: heightened parental mindfulness reduces parents' and children's psychopathology. Mindfulness 7, 680–689. 10.1007/s12671-016-0504-1 27217845 PMC4859846

[B32] Meyer J. M. Clapp J. D. Whiteside S. P. Dammann J. Kriegshauser K. D. Hale L. R.. (2018). Predictive relationship between parental beliefs and accommodation of pediatric anxiety. Behav. Therapy 49, 580–593. 10.1016/j.beth.2017.11.004 29937259

[B33] Möller E. L. MajdandŽić M. Bögels S. M. (2015). Parental anxiety, parenting behavior, and infant anxiety: differential associations for fathers and mothers. J. Child Fam. Stud. 24, 2626–2637. 10.1007/s10826-014-0065-7

[B34] Moreira H. Canavarro M. C. (2017). Psychometric properties of the Interpersonal Mindfulness in Parenting Scale in a sample of Portuguese mothers. Mindfulness 8, 691–706. 10.1007/s12671-016-0647-0

[B35] Moreira H. Canavarro M. C. (2018). The association between self-critical rumination and parenting stress: the mediating role of mindful parenting. J. Youth Adolesc. 49, 192–211. 10.1007/s10964-019-01133-9

[B36] Moretti M. M. Peled M. (2004). Adolescent-parent attachment: Bonds that support healthy development. Paediatr. Child Health 9, 551–555. 10.1093/pch/9.8.551 19680483 PMC2724162

[B37] Morris A. S. Criss M. M. Silk J. S. Houltberg B. J. (2017). The impact of parenting on emotion regulation during childhood and adolescence. Child Dev. Perspect. 11, 233–238. 10.1111/cdep.12238

[B38] Paquette D. (2004). Theorizing the father-child relationship: Mechanisms and developmental outcomes. Hum. Dev. 47, 193–219. 10.1159/000078723

[B39] Potharst E. S. Aktar E. Rexwinkel M. Rigterink M. Bögels S. M. (2017). Mindful with your baby: feasibility, acceptability and effects of a mindful parenting group training for mothers and their babies in a mental health context. Mindfulness 8, 1236–1250. 10.1007/s12671-017-0699-9 28989548 PMC5605590

[B40] Potharst E. S. Leyland A. Colonnesi C. Veringa I. K. Salvadori E. A. Jakschik M.. (2020). Does mothers' self-reported mindful parenting relate to the observed quality of parenting behavior and mother-child interaction? Mindfulness. 10.1007/s12671-020-01533-0. [Epub ahead of print]. 33193907 PMC7652703

[B41] Rutherford H. J. V. Wallace N. S. Laurent H. K. Mayes L. C. (2015). Emotion regulation in parenthood. Dev. Rev. 36, 1–14. 10.1016/j.dr.2014.12.008 26085709 PMC4465117

[B42] Suveg C. Shaffer A. Morelen D. Thomassin K. (2011). Links between maternal and child psychopathology symptoms: mediation through child emotion regulation and moderation through maternal behavior. Child Psychiatry Hum. Dev. 42, 507–520. 10.1007/s10578-011-0223-8 21484417

[B43] Wolk C. B. Caporino N. E. McQuarrie S. Settipani C. A. Podell J. L. Crawley S.. (2016). Parental Attitudes, Beliefs, and Understanding of Anxiety (PABUA): development and psychometric properties of a measure. J. Anxiety Disord. 39, 71–78. 10.1016/j.janxdis.2016.03.001 26970877 PMC4811694

[B44] Yap M. B. H. Pilkington P. D. Ryan S. M. Jorm A. F. (2014). Parental factors associated with childhood anxiety, depression and internalizing problems: a systematic review and meta-analysis. J. Affect. Disord. 175, 424–440. 10.1016/j.jad.2015.01.050 25679197

